# Cataloging Coding Sequence Variations in Human Genome Databases

**DOI:** 10.1371/journal.pone.0003575

**Published:** 2008-10-30

**Authors:** Hong-Hee Won, Hee-Jin Kim, Kyung-A Lee, Jong-Won Kim

**Affiliations:** 1 Samsung Biomedical Research Institute, Samsung Medical Center, Gangnam-Gu, Seoul, South Korea; 2 Department of Bio and Brain Engineering, Korea Advanced Institute of Science and Technology, Yuseong-Gu, Daejeon, South Korea; 3 Department of Laboratory Medicine and Genetics, Sungkyunkwan University School of Medicine, Samsung Medical Center, Gangnam-Gu, Seoul, South Korea; 4 Department of Laboratory Medicine, Yonsei University College of Medicine, Gangnam-Gu, Seoul, South Korea; Pasteur Institute, France

## Abstract

**Background:**

With the recent growth of information on sequence variations in the human genome, predictions regarding the functional effects and relevance to disease phenotypes of coding sequence variations are becoming increasingly important. The aims of this study were to catalog protein-coding sequence variations (CVs) occurring in genetic variation databases and to use bioinformatic programs to analyze CVs. In addition, we aim to provide insight into the functionality of the reference databases.

**Methodology and Findings:**

To catalog CVs on a genome-wide scale with regard to protein function and disease, we investigated three representative databases; the Human Gene Mutation Database (HGMD), the Single Nucleotide Polymorphisms database (dbSNP), and the Haplotype Map (HapMap). Using these three databases, we analyzed CVs at the protein function level with bioinformatic programs. We proposed a combinatorial approach using the Support Vector Machine (SVM) to increase the performance of the prediction programs. By cataloging the coding sequence variations using these databases, we found that 4.36% of CVs from HGMD are concurrently registered in dbSNP (8.11% of CVs from dbSNP are concurrent in HGMD). The pattern of substitutions and functional consequences predicted by three bioinformatic programs was significantly different among concurrent CVs, and CVs occurring solely in HGMD or in dbSNP. The experimental results showed that the proposed SVM combination noticeably outperformed the individual prediction programs.

**Conclusions:**

This is the first study to compare human sequence variations in HGMD, dbSNP and HapMap at the genome-wide level. We found that a significant proportion of CVs in HGMD and dbSNP overlap, and we emphasize the need to use caution when interpreting the phenotypic relevance of these concurrent CVs. Combining bioinformatic programs can be helpful in predicting the functional consequences of CVs because it improved the performance of functional predictions.

## Introduction

Unprecedented advancements in molecular technology and major initiatives such as the Human Genome Project and the International Haplotype Map Project have created a need for methods to interpret the myriad sequence variations in the human genome, and to catalog disease-causing mutations. This has led to the question of how to systematically collect and curate the variation data [1]. In addition, progress in sequencing technology has allowed mutational analysis of more than 10,000 genes in a single individual and has accelerated research on disease-associated allelic variations at the whole genome sequencing level [2]. The human genome is estimated to have up to 200,000 amino acid-substituting variations in the protein-coding sequences (CVs), also called nonsynonymous single nucleotide polymorphisms (nsSNPs) [3]. While substituting CVs may contribute to phenotype differences among individuals such as hair color, skin color [4], [5] and disease susceptibility [6]–[9], certain CVs (generally missense mutations) are known to cause highly-penetrant, Mendelian-inherited pathological conditions. Missense mutations account for approximately half of all allelic variants underlying inherited human diseases [10], [11]. As a result, it is vital that we correlate and attribute specific phenotypes to certain CVs. Database searches and bioinformatic analyses are the primary tools for this purpose. The three most representative databases are Human Gene Mutation Database (HGMD), Single Nucleotide Polymorphisms database (dbSNP), and Haplotype Map (HapMap). Each database has been developed with different features, for different purposes.

Several studies have investigated CVs with regard to human disease phenotypes; however, they most commonly involved a limited number of genes, such as those related to familial cancer syndromes [12], [13]. Since no studies have investigated genome-wide CVs in different reference databases, we cataloged CVs that occur in both HGMD and dbSNP, and characterized concurrent CVs using bioinformatics to provide unique insight into the optimal uses of those databases. We first prepared three different datasets of CVs extracted from HGMD, dbSNP, and HapMap (defined as CVM, CVS, and CVH, respectively). We then analyzed how the three datasets overlap, and characterized the CVs according to the patterns of amino acid substitutions. In addition, we performed functional predictions of CVs using selected *in silico* algorithms as well as a combinatorial approach that we developed using the Support Vector Machine (SVM). We found that combination improved the prediction performance of individual algorithms.

## Results

### Coding Sequence Variations Concurrent in dbSNP and HGMD

**Figure 1:**
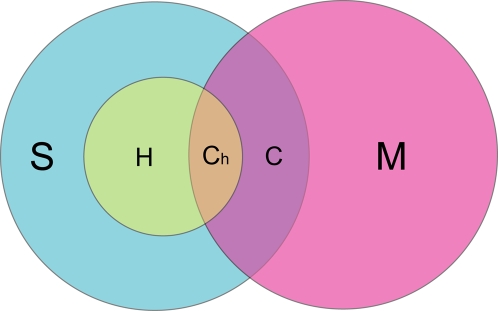
Comparison of variations of three databases. From the 1069 genes that have information on allelic variations both in dbSNPs and HGMD, a total of 12,399 CVS (group [S]) and 23,045 CVM (group [M]) are shown. Variations found in both dbSNP and HGMD are labeled ‘concurrent’ (group [C]). Variations occurring only in dbSNP or HGMD (mutually exclusive occurrence) are labeled [S-C] and [M-C], respectively. nsSNPs found in the HapMap database are labeled [H], and the concurrent group [C] is subdivided into [C_h_] (in HGMD and in HapMap) and [C-C_h_] (in HGMD and in dbSNP but not in HapMap).

A total of 74,784 CVs from 9513 genes were retrieved from the dbSNP database (CVS). A total of 26,037 CVs from 1477 genes were retrieved from the HGMD database (CVM) (Table 1). The average number of CVs per gene in dbSNP and HGMD was 7.9 and 17.6, respectively. The two datasets were then matched in a gene-specific manner to find CVs concurrent in the two databases. First, we obtained a list of 1069 genes that had sequence variation information in both dbSNP and HGMD. These ‘matched genes’ totaled 12,399 CVS (group [S] in Figure 1) and 23,045 CVM (group [M] in Figure 1). Among them, 1005 variations occurred both in dbSNP and in HGMD (‘concurrent’ CVs, group [C] in Figure 1; the complete list is in Table S1). In summary, 8.11% (1005/12,399) of the CVs from dbSNP are registered as CVM in HGMD, and 4.36% (1005/23,045) from HGMD were registered as CVS in dbSNP. We also made a subset of polymorphisms from a set of 12,399 CVS (group [S]) by a batch query to the HapMap dataset. As a result, we obtained a subset of 4414 CVH (group [H]), and a resultant subset of CVs that were concurrent in HapMap and in HGMD (group [C_h_] in Figure 1).

**Table 1:** Results of matching the SNP and mutation datasets.

Statistics	dbSNP	HGMD
No. of total genes	9513	1477
No. of total single base substitution variations	74,784	26,037
No. of matched genes	1069	
No. of variations of matched genes	12,399	23,045
No. of concurrent variations	1005	
Proportion of concurrent variations	8.11% (1005/12,399)	4.36% (1005/23,045)

Detailed distributions of CVs in the matched genes were analyzed and the resultant concurrent CVs sorted according to the type of variation (Table 2). Among the 12,399 CVS, nsSNPs accounted for the largest proportion (6809/12,399; 54.9%), followed by synonymous SNPs (snSNPs) (5416/12,399; 43.7%) and truncating SNPs (trSNPs) (174/12,399; 1.40%). The distribution was similar in the HapMap dataset (52.1%, 46.9%, and 1.02% for nsSNPs, snSNPs, and trSNPs, respectively). Among the 23,045 CVM, missense mutations accounted for the largest proportion (17,970/23,045; 78.0%), followed by nonsense mutations (5006/23,045; 21.7%). In a very small proportion of CVM (69/23,045; 0.299%), a nucleotide substitution was expected to retain the original amino acid, instead of changing it to another amino acid or stop codon. Among the concurrent CVs (group [C]), amino acid-substituting (AA1>AA2) CVs were by far the most common (934/1005; 92.9%). Truncation variations accounted for 4.08% (41/1005), followed by variations expected to retain the original amino acid (30/1005; 2.99%). When CVH were taken into consideration, the proportion of amino acid-substituting CVs was almost unchanged (302/332; 91.0%). The proportion of the AA1>AA1 group (20/332; 6.02%) was higher than that of truncation variations (10/332; 3.01%). We observed that variations in three different categories in the concurrent CVs (group [C]) showed different distributions of SNP validation scores calculated from frequency information, submitter's validation and availability in HapMap (Validation status code is available at ftp://ftp.ncbi.nih.gov/snp/database/organism_shared_data/SnpValidationCode.bcp.gz) (Figure S1). The average of trSNPs validation scores was the lowest at 1.27, followed by the nsSNPs at 2.03. The snSNPs average was the highest at 6.40. Among the 41 trSNPs, validation scores of 33 variations (80.5%) were less than 3, while among the 30 snSNPs, validation scores of 23 variations (76.7%) were greater than or equal to 3. The percent of variations of validation score greater than 9 was 7.32% (3/41) for the trSNPs and 35.5% (11/31) for the snSNPs. These results indicate that most trSNPs in dbSNP were relatively less validated when compared to snSNPs in dbSNP.

**Table 2:** Single amino acid-substituting variations and distribution of concurrent variations in dbSNP/HapMap and HGMD by type of variation.

Type of variation	AA1>AA2^a^	AA1>TR^b^	AA1>AA1^c^	Total	Figure 1 d
dbSNP (No./%)	6809 (54.9)	174 (1.40)	5416 (43.7)	12,399 (100)	S
HapMap (No./%)	2299 (52.1)	45 (1.02)	2070 (46.9)	4414 (100)	H
HGMD (No./%)	17,970 (78.0)	5006 (21.7)	69 (0.30)	23,045 (100)	M
dbSNP∩HGMD^e^ (No./%)	934 (92.9)	41 (4.08)	30 (2.99)	1005 (100)	C
dbSNP∩HapMap∩HGMD^f^ (No./%)	302 (91.0)	10 (3.01)	20 (6.02)	332 (100)	C_h_

a nsSNP or missense mutation: variation expected to replace one amino acid (AA1) with a different amino acid (AA2)

b trSNP or nonsense mutation: variation expected to replace one amino acid (AA1) with a termination codon (TR)

c snSNP: variation expected not to change the original amino acid (AA1)

d Variation groups represented in Figure 1 diagram

e Variations concurrent in dbSNP and HGMD

f Variations concurrent in dbSNP, HapMap, and HGMD

Since the issue of distinction between neutral and damaging variations is frequently addressed for cancer-predisposing genes, we focused our analyses on 86 genes associated with well-known hereditary cancer syndromes [14] among the 1069 matched genes. We found that 29 genes (33.7%) had at least one concurrent CV (Table S2). The total number of concurrent CVs encountered in those 29 genes was 141, which accounted for 14.0% of the total concurrent CVs (Table S3). The largest number of concurrent CVs was observed in the *VHL* gene (von Hippel-Lindau syndrome, MIM# 193300), followed by *BRCA1*/*BRCA2* (hereditary breast cancer syndrome, MIM# 113705, 600185). The number of nsSNPs was greater than that of missense mutations in four genes (*FANCC*, *WRN*, *PMS2*, and *CDK4*).

### Pattern of Amino Acid Substitutions According to Amino Acid Group

**Figure 2:**
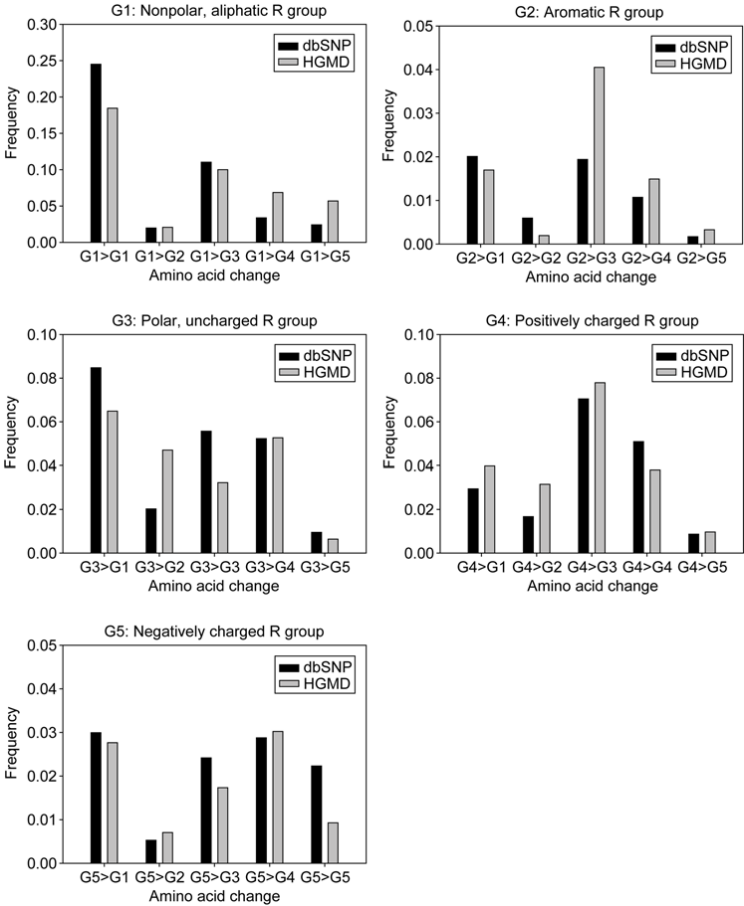
Distribution of amino acid change frequencies according to amino acid group (G1, nonpolar, aliphatic R group; G2, aromatic R group; G3, polar, uncharged R group; G4, positively charged R group; and G5, negatively charged R group). Black vertical bars indicate group [S-C] in dbSNP and gray vertical bars indicate group [M-C] in HGMD.

We analyzed the pattern of amino acid substitutions in three disjointed groups of variations, [S-C], [M-C], and [C], according to five amino acid groups (G1, nonpolar, aliphatic R group; G2, aromatic R group; G3, polar, uncharged R group; G4, positively charged R group; and G5, negatively charged R group). The most striking difference between groups [S-C] and [M-C] was observed in amino acid substitutions from the aromatic R group (G2) to the polar, uncharged R group (G3) (1.9% vs 4.1%; see Figure 2). In addition, substitutions from the polar, uncharged R group (G3) to the aromatic R group (G2) were observed more often in group [M-C] than in group [S-C] (4.7% vs 2.0%). To measure the difference between the two types of variations, we calculated the chi-squared values with degrees of freedom = 1. A significant difference was found (*X* ^2^ = 143.8, *P*<3.9×10^−33^) between group [S-C] and group [M-C] in the frequency of G2-G3 substitutions. This result suggests the transition of amino acids between G2 and G3 could potentially exert deleterious effects on protein function. Differences in the frequency of G2-G4 and G1-G4 substitutions were notably high (*X* ^2^ = 39.7, *P*<3.0×10^−10^ and *X* ^2^ = 102.2, *P*<5.2×10^−24^, respectively). In contrast, amino acid substitutions between G3 and G5 occurred in group [S-C] slightly more often than in group [M-C] (3.4% vs 2.3%). Remarkably, in all amino acid groups, substitutions within the same amino acid group were more frequently observed in group [S-C] than in group [M-C]. We have summarized these results in Table 3, based on two types of amino acid substitutions—those within the same amino acid group and those between different groups. The proportion of amino acid substitutions within the same group in group [S-C] was larger than in group [M-C] (38.0% vs 26.6%). For example, the frequency of substitutions from valine to isoleucine in the nonpolar, aliphatic R group was 2.4% in group [S-C], but 0.6% in group [M-C]. Similarly, the frequency of substitutions from isoleucine to valine in the nonpolar, aliphatic R group was 1.8% in group [S-C], while it was 0.4% in group [M-C]. This finding indicates that an amino acid substitution in an nsSNP may be less likely to change the function of a protein. Substitutions within the same amino acid group accounted for 30.8% of the concurrent variants (group [C]), which falls between those in group [S-C] and in group [M-C] (Table 3). The pattern of amino acid substitutions in group [S-C] was significantly different from that in group [M-C] (*X* ^2^ = 417.3, *P*<9.5×10^−93^), which might reflect the different underlying mutagenic mechanisms that create the variations in these two groups. The pattern showed a significant difference between group [C] and group [M-C] (*X* ^2^ = 203.8, *P*<3.1×10^−46^), than between group [C] and group [S-C] (*X* ^2^ = 17.7, *P*<2.6×10^−5^).

**Table 3:** Comparison of variations according to type of amino acid substitution.

	nsSNPs in dbSNP	Missense mutations in HGMD	Concurrent variations
Substitution within the same amino acid group	2232 (38.0%)	4534 (26.6%)	288 (30.8%)
Substitution between two different amino acid groups	3643 (62.0%)	12,502 (73.4%)	646 (69.2%)

### Functional Prediction of Amino Acid-Substituting Variations by Different *In Silico* Tools

Efforts have been made to develop algorithms for the prediction of functional impact of a known or novel amino acid-substituting variation without experimental characterization [15]–[22]. For the functional prediction of amino acid-substituting CVs, which accounted for the largest number of variations both in dbSNP (nsSNPs) and in HGMD (missense mutations), as well as in the group of concurrent variations (group [C] and [C_h_]), we employed three widely-used prediction programs—Sorting Intolerant From Tolerant (SIFT) [18], Polymorphism Phenotyping (PolyPhen) [19] and Protein Analysis Through Evolutionary Relationships (PANTHER) [21]. Two datasets were used, a set of 5875 nsSNPs in group [S-C] (6809 minus 934) from dbSNP, and a set of 17,036 missense mutations in group [M-C] (17,970 minus 934) from HGMD (Figure 1 and Table 2). We also used nsSNPs in HapMap (group [H-C_h_]), since HapMap is generally regarded to contain neutral or less penetrant variations than dbSNP. SIFT and PANTHER give results in two prediction categories—tolerated or deleterious effects, while PolyPhen gives results in three categories—benign (probably lacking any phenotypic effect), possibly damaging, and probably damaging (should affect protein function) [19]. Prediction results are given as unknown when the reference data are insufficient. We excluded variations with ambiguous prediction results from the analyses.

From the HapMap dataset (group [H-C_h_]), PANTHER and SIFT predicted that 72.2% and 69.8% of the tested nsSNPs, respectively, would be tolerated, and PolyPhen predicted 66.3% of the tested nsSNPs to be benign (Table 4). The prediction results for nsSNPs from dbSNP (group [S-C]) were slightly different from those for nsSNPs from HapMap (group [H-C_h_]). The proportion of nsSNPs from dbSNP predicted to be deleterious was larger than that of nsSNPs from HapMap (36.3% vs 30.2% for the SIFT prediction results and 33.7% vs 27.8% for the PANTHER prediction results). Likewise, the proportion of nsSNPs from dbSNP predicted to be damaging by Polyphen was larger than that of nsSNPs from HapMap (36.9% vs 33.7%). When we analyzed the damaging effects predicted by PolyPhen, the proportion of variations predicted to be ‘possibly’ damaging was larger than that predicted to be ‘probably’ damaging in the HapMap dataset (19.1% vs 14.6%), while the proportions were almost the same in the dbSNP dataset (18.0% vs 18.9%). In the HGMD dataset, the majority of missense mutations were predicted to be deleterious by SIFT (65.2%) and PANTHER (70.6%), and to be damaging by Polyphen (73.6%). In addition, the proportion of missense mutations predicted to be probably damaging was almost twice that predicted to be possibly damaging (49.7% vs 23.8%).

**Table 4:** Predicted phenotypic effects of amino acid substitutions from three datasets by SIFT, PolyPhen, and PANTHER.

Datasets	Prediction of phenotypic effect of amino acid substitutions						
	SIFT		PolyPhen			PANTHER	
	Tolerated	Deleterious	Benign	Possibly damaging	Probably damaging	Tolerated	Deleterious
nsSNPs in HapMap ([H-C_h_])	69.8% (1005/1439)	30.2% (434/1439)	66.3% (811/1223)	19.1% (234/1223)	14.6% (178/1223)	72.2% (782/1083)	27.8% (301/1083)
nsSNPs in dbSNP ([S-C])	63.7% (2793/4387)	36.3% (1594/4387)	63.1% (2560/4056)	18.0% (730/4056)	18.9% (766/4056)	66.3% (2290/3453)	33.7% (1163/3453)
Missense mutations in HGMD ([M-C])	34.8% (4516/12,995)	65.2% (8479/12,995)	26.4% (2922/11,049)	23.8% (2635/11,049)	49.7% (5492/11,049)	29.4% (2727/9265)	70.6% (6538/9265)

Assuming that the HapMap dataset mainly comprises non-pathogenic variations (negative samples) and the HGMD dataset comprises pathogenic variations (positive samples), we compared the prediction performances of SIFT, PolyPhen, and PANTHER using several criteria including sensitivity, specificity, positive predictive value (p.p.v.), the Pearson correlation coefficient, and true-positive cost. PANTHER showed the best prediction performance among the three prediction programs. PANTHER surpassed SIFT in all criteria and PolyPhen in specificity, p.p.v., and true-positive cost (Table 5). PolyPhen surpassed SIFT in all criteria except for specificity. PolyPhen was particularly superior to SIFT in sensitivity (73.6% vs 65.2%, or 8.4%), which indicates that PolyPhen predicts damaging CVs more accurately than SIFT. The difference in specificity between SIFT and PolyPhen was 3.5%.

**Table 5:** Comparison of the prediction performance of SIFT, PolyPhen, and PANTHER.

Performance measure criterion	SIFT	PolyPhen	PANTHER
Sensitivity (%)^a^	65.2	73.6	70.6
Specificity (%)^b^	69.8	66.3	72.2
p.p.v. (%)^c^	95.1	95.2	95.6
Correlation coefficient^d^	2.31	3.12	2.70
True-positive cost^e^	0.0512	0.0507	0.0460

a Sensitivity = true positive/(true positive+false negative)

b Specificity = true negative/(true negative+false positive)

c Positive predictive value = true positive/(true positive+false positive)

d Correlation coefficient = (true positive×true positive−false positive×false negative)/((true positive+false positive) (true positive+false negative) (true negative+false positive)(true negative+false negative))^1/2^

e True-positive cost = false positive/true positive

### Combination of the Prediction Programs

**Figure 3:**
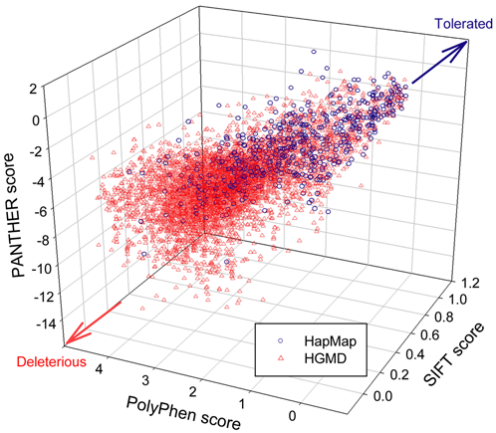
Scatter plot of predicted scores of SIFT, PolyPhen, and PANTHER. Red triangles and blue circle plots indicate missense mutations and nsSNPs, respectively. The ranges of predicted scores of the three programs were different from each other (0–1 for SIFT, 0–4.6 for PolyPhen, and -13.4–1.1 for PANTHER). Lower SIFT or PANTHER scores, or higher PolyPhen scores indicate that the predicted variant is more deleterious. A close distribution in the left lower corner represents an agreement of the three programs on deleterious effect.

Predictions of the functional consequences of amino acid substitutions can be made more accurate by combining different *in silico* methods [15]. Thus, we combined prediction programs, and found that this could significantly increase prediction performance. Before combining the programs, we determined the differences in the predictions of each program, since the combination would be of no significant use if individual programs produced identical predictions. Figure 3 shows a scatter plot of the predicted scores of SIFT, PolyPhen, and PANTHER. Since a lower SIFT or PANTHER score and a higher PolyPhen score indicate that the variation of interest would be more deleterious, a close distribution of the variation near the left lower corner demonstrates agreement between the three programs on the prediction. Missense mutations in CVM tended toward the left lower corner (deleterious), while nsSNPs in CVH tended to be in the right upper corner (tolerated). Even though HapMap and HGMD tended to go in opposite directions, no significant dependencies were observed in the distribution of prediction scores by the three programs. The independence between the predictions suggests a possibility that the combination of the programs could improve the prediction performance.

**Figure 4:**
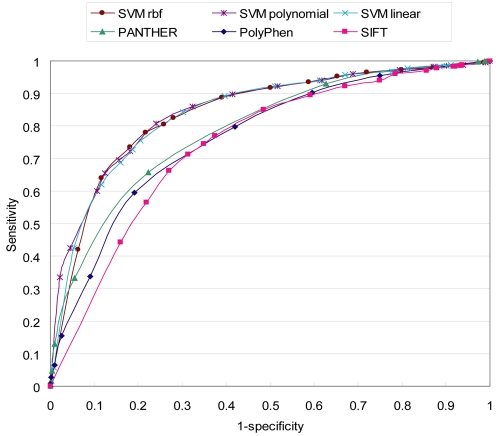
Receiver Operating Characteristic (ROC) curves of the combination and individual predictors. The ROC curves of SVM combination predictors tend toward the upper left corner of the plot more than the three individual prediction programs. This suggests that SVM combinations outperform the individual prediction programs. SVM_polynomial_ showed a specificity of 75.9% and a sensitivity of 80.7% when the threshold of SVM combination score was 0.8 (see Table 5).

To evaluate the combination approach, we regarded all of the CVH data as negative and the CVM data as positive. We used the SVMs of three different kernel functions in combining the three programs. To train SVM, we randomly chose 281 of 563 (50%) CVH from HapMap and 562 of 6282 (8.9%) CVM from HGMD as a training dataset. The three kernels used for SVM training were a linear kernel, a polynomial kernel, and a radial basis function kernel. We evaluated the prediction performance of the three models with the testing dataset. As shown in Figure 4, the SVM combination outperformed the individual prediction programs. SVM_polynomial_ showed a slightly better prediction performance in the region of high specificity than the other two SVM combinations. PANTHER was superior to PolyPhen and SIFT in terms of sensitivity over all specificity regions. SVM_polynomial_ generally gave accurate and stable predictions compared to the other combination approaches and the individual programs, indicating that the appropriate combination can noticeably improve the prediction accuracy.

## Discussion

In this study, we performed a genome-wide analysis of variations in the coding sequence of the human genome in different reference databases. CVM data from HGMD mainly include CVs that are causative or highly penetrant for a particular inherited disorder [23], while CVS from dbSNP and CVH from HapMap mainly include CVs that are neutral or of low penetrance. Therefore, CVs from different databases should show different characteristics in several aspects such as amino acid substitutions, protein function changes, and allele frequencies. This is confirmed by the results of this analysis.

We observed that up to 8.11% of CVS in dbSNP are registered as CVM in HGMD, and 4.36% of the CVM in HGMD are registered as CVS in dbSNP. Among these concurrent CVs, there were 41 truncation variations, 10 of which were also found in the HapMap database. Some of the truncation CVs with low validation scores (Figure S1) were found to be disease-causing mutations. Heterozygosity information was available for 7 variations out of the 41. When we examined these seven concurrent CVs, two of them appeared to be nonsense mutations. The mutant alleles occurred in normal controls at relatively high frequencies due to founder effects, for example, rs17602729 or Gln12Term in the *AMPD1* gene causing adenosine monophosphate deaminase deficiency [24], rs12948217 or Tyr231Term in the *ASPA* gene causing Canavan diseases [25], and rs11571833 or Lys3326Term in the *BRCA2* gene causing hereditary breast and ovarian syndrome. The remaining CVs include apparently deleterious mutations at the molecular level, but their phenotypic effects, *i.e.*, disease association, should be interpreted with caution. This is particularly the case for truncation CVs in the pharmacogenetics genes and human leukocyte antigens (HLA) (rs4986893 or Trp212Term in the *CYP2C19* gene [26] and rs1131156 or Ser14Term in the *HLA-B* gene [27]). Thirty snSNPs were concurrent in HGMD, and 20 of them were also found in the HapMap database. When we reviewed the references linked to these 20 variations at HGMD, we found that most of them were snSNPs and were registered in the HGMD database since they were found to be associated with susceptibility mainly to common complex diseases such as diabetes (see reference links in Table S1). These observations indicate that some SNPs in dbSNP are truly phenotypically mutational, and the interpretation of their functional consequences should be based on the context of carriers in the general population (or specific populations in case of founder mutations), or in the context of the phenotypic impact of the mutant molecule (for example, drug metabolism). In addition, some truncation CVs need to be considered in the same context as copy number variations, which have been recently reported to be more common in the human genome than previously recognized [28]. Some mutations in HGMD may be disease susceptibility SNPs with low penetrance or in linkage to other risk alleles. Although we examined the individual variations for these two types of concurrent variations only, that is, truncation mutations and snSNPs, our observations may be valid for at least some of the amino acid-substituting variations in the human genome that accounted for more than 90% of concurrent CVs. One of the best examples is the factor V Leiden mutation in the *F5* gene (R506Q, rs6020), which is a founder mutation in Caucasian populations [29]. The factor V Leiden mutation results in activated protein C resistance at the molecular level and predisposes mutation carriers to develop thrombotic diseases. Other examples include ABO blood group variations.

In a particular set of 29 genes related to major cancer predisposition syndromes, we also observed 141 concurrent CVs. Among the 14 truncation variations concurrent in dbSNP and HGMD, 10 occurred in the *VHL* gene. Von Hippel Lindau syndrome is an autosomal dominant disease and thus one mutant allele can cause the disease phenotype. To investigate the variations of *VHL* in the general population, we resequenced 192 control chromosomes of Korean ethnic origin but found no variation. Although we did not examine control chromosomes of other ethnic origins, we believe that the truncation variations in dbSNP of *VHL* are nonsense mutations.

Some genes with leader peptide sequences that are removed in mature proteins have codon numbers based on the mature protein in HGMD, rather than having the first Met as +1 according to the recommendations by the Human Genome Variation Society (http://www.hgvs.org/mutnomen/), for example, the coagulation factor proteins such as *F5*. For these genes, one should take into account the leader peptide sequences when examining the current mutation database. Indeed, we could not match dbSNP and HGMD for these genes because of their different codon numbering, which explains why we could not find the aforementioned factor V Leiden mutation in our catalog of concurrent CVs.

In the distribution of amino acid substitutions, the most remarkable difference between groups [S-C] and [M-C] was observed in amino acid substitutions between the aromatic R group (G2) and the polar, uncharged R group (G3). The substitutions between G2 and G3 occurred in 8.8% of total variations in group [M-C] and 3.9% in group [S-C]. The substitution frequency agrees well with the chemical distance (CD) from Grantham's chemical difference matrix [30] (Table S4). Relatively frequent substitutions in group [M-C] tend to have long distances between amino acids, whereas relatively frequent substitutions in group [S-C] tend to have short distances between two amino acids. The substitution from cysteine (G2) to phenylalanine (G3) occurred four times more frequently in group [M-C] than in group [S-C], and the CD between cysteine and phenylalanine was 205. For reference, the CDs of all amino acid pairs range from 5 to 215. The cysteine (G2)-to-tyrosine (G3) substitution occurred ∼10 times more often in group [M-C] than in group [S-C], and the CD between cysteine and tyrosine was also high (CD = 194). The substitution between the most chemically distant amino acid pair (cysteine [G2] and tryptophan [G3]) occurred three times more often in group [M-C] than in group [S-C]. Conversely, the substitution between isoleucine (G2) and valine (G2) was observed three times more often in group [S-C] than in group [M-C], and the CD between isoleucine and valine is five. The threonine (G3)-to-serine (G3) substitution occurred seven times more frequently in group [S-C] than in group [M-C], and the CD between threonine and serine is 58. Remarkablely, in all the amino acid groups, substitutions within the same amino acid group were observed more frequently in group [S-C] than in group [M-C] (38.0% vs 26.6%). Substitutions within the same amino acid group accounted for 30.8% in the concurrent CVs (group [C]), which fell between those in group [S-C] and in group [M-C]. This observation suggests that the concurrent CVs might be a mixture of SNPs and mutations, supporting the hypothesis that some CVS in dbSNP are truly phenotypic mutations, and some CVM in HGMD are disease-susceptibility SNPs.

We used the SIFT, PolyPhen, and PANTHER programs to predict the influence of thousands of amino acid-substituting CVs on protein function. The three programs predicted 34–37% of the nsSNPs in dbSNP and 28–34% of the nsSNPs in HapMap to be deleterious or damaging. By other amino acid substitution programs, approximately 25–30% of the putative nsSNPs cataloged in dbSNP were predicted to negatively affect the protein function [15]. These tend to be rare in the population. Among the nsSNPs in dbSNP predicted to be deleterious, nsSNPs that are not in HapMap need to be validated by experimental methods because they might be rare variations that contribute to disease phenotypes.

A performance evaluation showed that PANTHER gave better specificity, p.p.v., and true-positive cost than PolyPhen and SIFT, while PolyPhen was better for sensitivity and correlation coefficient than PANTHER and SIFT. In contrast, PANTHER produced unpredicted results more often than the other two programs. The non-prediction rate of PANTHER ranged from 41.2% to 45.8%, the non-prediction rates of PolyPhen and SIFT ranged from 31.0% to 38.8% and 23.7% to 27.9%, respectively (PANTHER: 45.8% for group [H-C_h_], 41.2% for group [S-C] and 45.6% for group [M-C]; PolyPhen: 38.8% for group [H-C_h_], 31.0% for group [S-C] and 35.1% for group [M-C]; SIFT: 27.9% for group [H-C_h_], 25.3% for group [S-C] and 23.7% for group [M-C]). The high non-prediction rate probably because PANTHER uses the source of sequences and structures more strictly than PolyPhen and SIFT and the reference data are not sufficient. The insufficient prediction rate will be overcome as more information is available [15]. It might be helpful to utilize a number of locus-specific mutation databases for specific diseases or genes such as the HbVar database [31] for human hemoglobin variations and thalassemia mutations, and the IDR database [32] for immunodeficiency mutations.

Lastly, we could significantly increase the prediction power by combining the programs, and we hypothesize that combining more prediction programs may further improve the prediction performance [16], [17], [20], [22]. In the HGMD dataset, more missense mutations were predicted to be deleterious by the SVM_polynomial_ combination than nsSNPs from dbSNP. This result supports the hypothesis that the functional prediction of CVs could distinguish between missense mutations underlying Mendelian diseases and functionally neutral nsSNPs [15]. In particular, the largest proportion of CVs (326/461; 70.7%) in the concurrent group [C] was predicted to be damaging (Table S5). This might include a large fraction of medically detrimental mutations that are not eliminated by purifying selection, possibly due to positive selection or mutation-selection balance [33]. For variations in group [C_h_], the proportion of variations predicted to be damaging was high (63/135; 46.7%), and the average allele frequency was relatively low, compared to those predicted to be neutral (Figure S2). The T-test result showed that the differences in allele frequencies between the two variations for each population were significant (*P*<0.05). We also found that some variations predicted to be damaging, such as rs16941 and rs2020873, showed strong evidence for positive selection (either an integrated haplotype score >2 or <−2 [34]) (Table S5). Evidence of positive selection may be helpful in identifying genetic variations associated with complex disease [35]. These results suggest that some concurrent variations predicted to be damaging could be nsSNPs that affect protein function less severely than missense mutations. Some common complex diseases may be caused by a multitude of rare variations that continue to exist because of an evolutionary mechanism [33].

In summary, this is the first study to look into human sequence variations genome-wide in three representative reference databases HGMD, dbSNP and HapMap. We found that a significant proportion of CVs in HGMD and dbSNP overlap, and particular caution is required in assessing these concurrent CVs with regard to their phenotypic relevance. Bioinformatic analyses that employ a combination of different algorithms can be helpful in determining genotype-phenotype correlations by providing improved functional predictions.

## Materials and Methods

### Reference Databases

We used three representative human sequence variation databases: HGMD (http://www.hgmd.cf.ac.uk/ac/index.php; public version January 2006), dbSNP (http://www.ncbi.nlm.nih.gov/projects/SNP/; build 126), and HapMap (www.hapmap.org; NCBI build 35). In general, “damaging and deleterious variations” indicate variations underlying highly penetrant diseases, and “neutral or susceptibility variations” indicate variations known to be phenotypically silent or associated with increased risk of developing a disease [36]. The HapMap database is a subset of the dbSNP database and is less likely to include pathogenic variants, since the HapMap data were generated from randomly selected individuals.

### Tools for Predicting Phenotypic Effects of Amino Acid Substitution

We applied three representative *in silico* programs, SIFT [18], PolyPhen [19], and PANTHER [21], to predict the phenotypic effect of a CV amino acid substitution. The three programs use different algorithms and thus may give a different prediction for the same CV. As reference protein database, we used SWISS-PROT for SIFT, PQS for PolyPhen, and PANTHER 6.1 for PANTHER. To set up SIFT and PANTHER locally, we downloaded SIFT (Linux version 2.1) from http://blocks.fhcrc.org/sift/SIFT.html, PANTHER SNP scoring tool (Linux version 1.0) from http://www.pantherdb.org/downloads and SWISS-PROT from ftp://ftp.ncbi.nih.gov/blast/db/FASTA. We executed PolyPhen online (http://genetics.bwh.harvard.edu/pph) through batch processing, and have summarized the results.

### Combination Analysis using SVM

By estimating the discriminant function, SVM classifies the data into two classes [37]. It builds up a hyperplane as the decision surface in a way that maximizes the margin of separation between positive and negative examples. Given a labeled set of *M* training samples (***X_i_***, ***Y_i_***), where ***X_i_***∈*R^N^* and ***Y_i_*** is the associated label, ***Y_i_***∈−1, 1, the discriminant hyperplane is defined by:
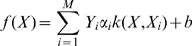
(1)where *k*(·,·) is a kernel function and the sign of *f*(*X*) determines the membership of *X*. In our problem, ***X_i_*** is a three-dimensional vector (***x_i1_***, ***x_i2_***, ***x_i3_***) where ***x_i1_*** is the SIFT score, ***x_i2_*** is the PolyPhen score and ***x_i3_*** is the PANTHER subPSEC score of the variant of interest, and ***Y_i_*** indicates the variant is positive (damaging CV) or negative (neutral CV). Because the original prediction scores of each program are on different scales (0–1 for SIFT, 0–4.6 for PolyPhen, and −13.4–1.1 for PANTHER), we used the following equation to make the score fall within the 0–1 range:
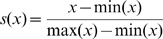
(2)where *s*(*x*) is a scaled score of an original prediction score *x*, and min(*x*) and max(*x*) are the minimum and maximum values of *x*, respectively.

We defined the SIFT and PANTHER scores as 1 minus their scaled prediction value because a prediction value close to 0 means a deleterious variant and a value close to 1 means a tolerant variant. We used the SVM^light^ software package [38], which is available at http://svmlight.joachims.org.

## Supporting Information

Figure S1 Validation scores of concurrent variations (group [C]) according to variation type. Validation scores were obtained from dbSNP. Horizontal bold lines show the average validation score. The average validation score of nsSNPs (AA1>AA2) was 2.03, the average of trSNPs (AA1>TR) was 1.27, and the average of snSNPs (AA1>AA1) was 6.40 (see Table S1).
(6.19 MB TIF)

Figure S2 Distribution of the variant allele frequency observed in the three populations (CEU: Caucasian; ASN: Asian; and YRI: African) in HapMap for variants in group [C_h_] (for details, see Table S5). The horizontal bold line in each population shows the average variant allele frequency. For 63 variants predicted to be damaging by the SVM combination, the average variant allele frequency is relatively low (CEU: 0.09; ASN: 0.07; and YRI: 0.06), compared to 72 variants predicted to be neutral by the SVM combination (CEU: 0.15; ASN: 0.15; and YRI: 0.17). The difference between the two variant groups was significant based on the result of the t-test.
(6.43 MB TIF)

Table S1 (0.51 MB XLS)

Table S2 (0.04 MB DOC)

Table S3 (0.09 MB XLS)

Table S4 (0.03 MB XLS)

Table S5 (0.31 MB XLS)
