# Supplementary figures and images for: Cataloging Coding Sequence Variations in Human Genome Databases
Source: PLoS One. 2008 Oct 30;3(10):e3575. doi: 10.1371/journal.pone.0003575 (PMC2570488; doi:10.1371/journal.pone.0003575)

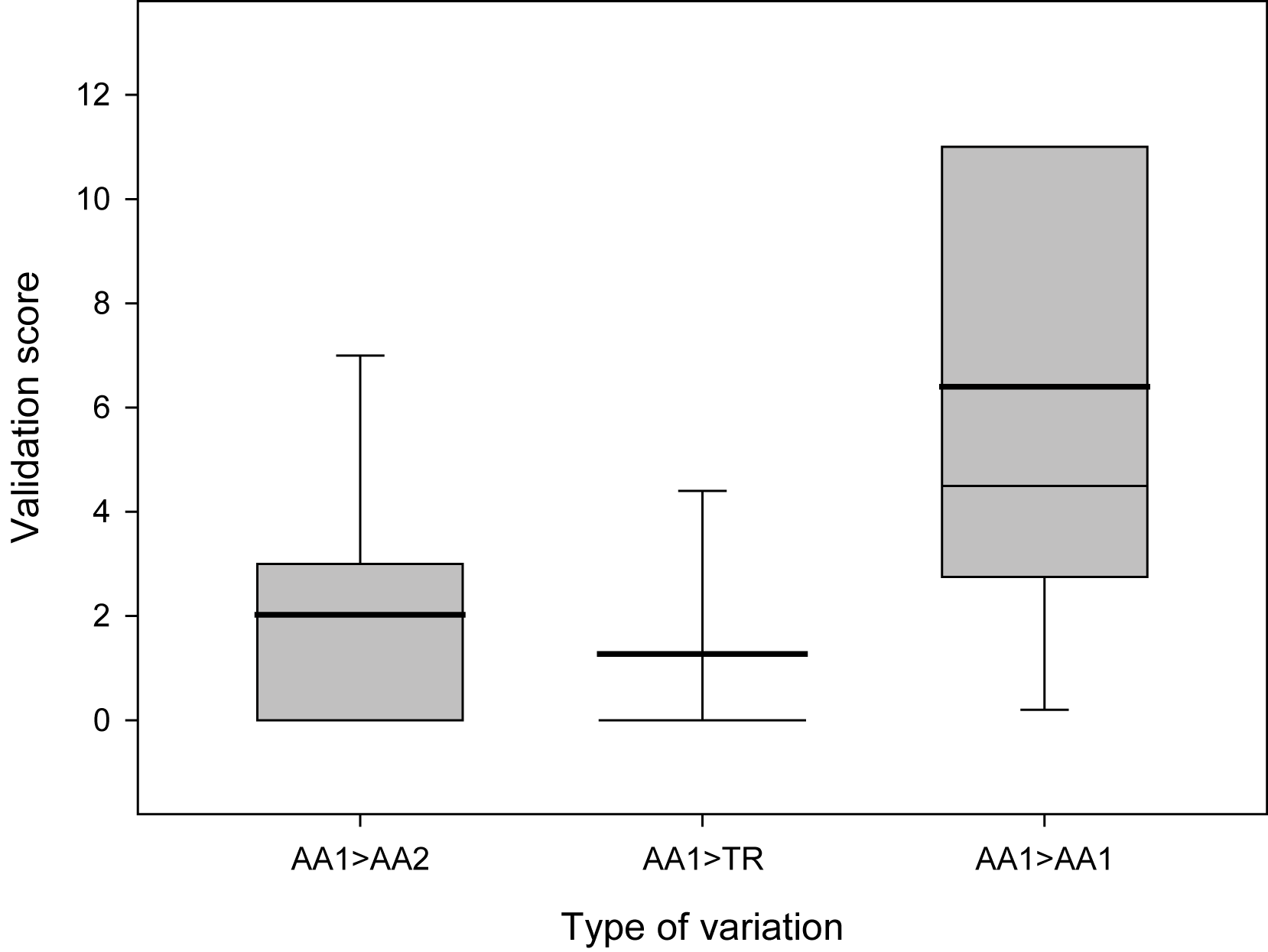

Supplement: Figure S1 — Validation scores of concurrent variations (group [C]) according to variation type. Validation scores were obtained from dbSNP. Horizontal bold lines show the average validation score. The average validation score of nsSNPs (AA1>AA2) was 2.03, the average of trSNPs (AA1>TR) was 1.27, and the average of snSNPs (AA1>AA1) was 6.40 (see Table S1). (6.19 MB TIF) [file pone.0003575.s001.tif]

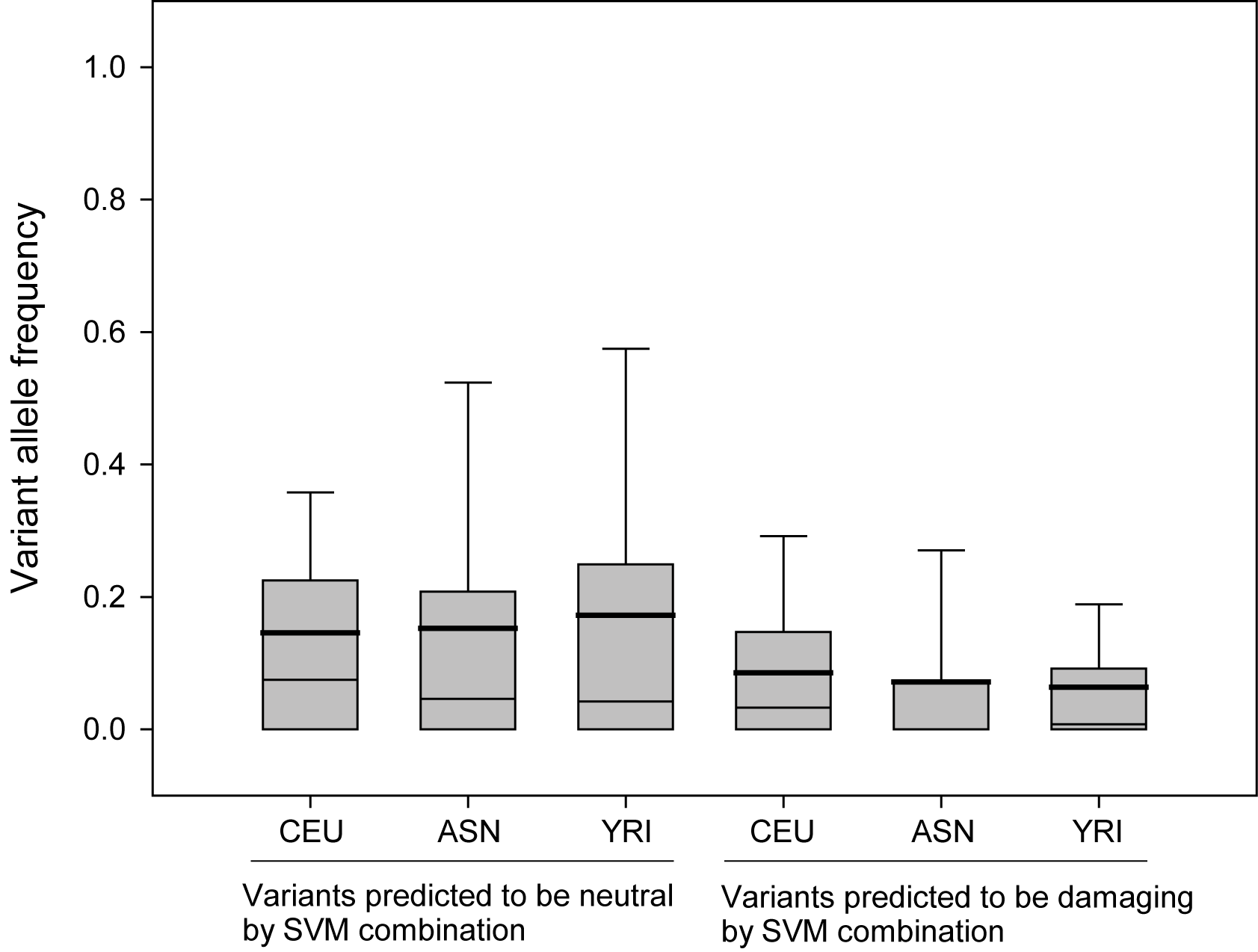

Supplement: Figure S2 — Distribution of the variant allele frequency observed in the three populations (CEU: Caucasian; ASN: Asian; and YRI: African) in HapMap for variants in group [Ch] (for details, see Table S5). The horizontal bold line in each population shows the average variant allele frequency. For 63 variants predicted to be damaging by the SVM combination, the average variant allele frequency is relatively low (CEU: 0.09; ASN: 0.07; and YRI: 0.06), compared to 72 variants predicted to be neutral by the SVM combination (CEU: 0.15; ASN: 0.15; and YRI: 0.17). The difference between the two variant groups was significant based on the result of the t-test. (6.43 MB TIF) [file pone.0003575.s002.tif]
