# Supplementary material for: Cataloging Coding Sequence Variations in Human Genome Databases
Source: PLoS One. 2008 Oct 30;3(10):e3575. doi: 10.1371/journal.pone.0003575 (PMC2570488; doi:10.1371/journal.pone.0003575)
Supplement: Table S2 — (0.04 MB DOC) [file pone.0003575.s004.doc]

Table S2. Concurrent variations in 29 genes associated with hereditary cancer syndromes

| **Syndrome (OMIM entry)** | **Gene** | **No. of SNPs** | **No. of mutations** | **No. of concurrent variations**  **(AA1>AA2^a^/TR^b^/AA1^c^)** |
| --- | --- | --- | --- | --- |
| von Hippel-Lindau syndrome (193300) | *VHL* | 42 | 164 | 35(25/10/0) |
| Hereditary breast cancer syndromes (113705, 600185) | *BRCA1* | 88 | 206 | 17(17/0/0) |
|  | *BRCA2* | 116 | 122 | 20(19/1/0) |
| Li-Fraumeni Syndrome (151623) | *TP53* | 26 | 90 | 11(11/0/0) |
|  | *CHEK2* | 16 | 19 | 1(1/0/0) |
| Ataxia telangiectasia (208900) | *ATM* | 88 | 137 | 9(9/0/0) |
| Fanconi anemia (227650) | *FANCA* | 33 | 41 | 6(6/0/0) |
|  | *FANCG* | 8 | 15 | 3(3/0/0) |
|  | *FANCC* | 11 | 7 | 1(1/0/0) |
| Endocrine cancer predisposition syndromes, MEN2 (171400) | *RET* | 12 | 140 | 5(3/0/2) |
| Hereditary gastrointestinal malignancies (120435, 120426, 114500, 114400) | *MLH1* | 26 | 138 | 4(4/0/0) |
|  | *MSH2* | 14 | 115 | 4(4/0/0) |
|  | *MSH6* | 22 | 35 | 2(2/0/0) |
| Familial polyposis (175100) | *APC* | 22 | 176 | 3(3/0/0) |
| Hereditary melanoma pancreatic cancer syndrome (606719) | *CDKN2A* | 7 | 54 | 3(3/0/0) |
| Retinoblastoma (180200) | *RB1* | 12 | 92 | 3(2/1/0) |
| Werner’s Syndrome (277700) | *WRN* | 53 | 8 | 2(1/1/0) |
| Turcot Syndrome (276300) | *PMS2* | 20 | 3 | 1(1/0/0) |
| Familial gastrointestinal stromal tumor (606764) | *KIT* | 8 | 25 | 1(1/0/0) |
| Melanoma syndromes (155600, 155601, 609048, 608035) | *CDK4* | 13 | 4 | 1(1/0/0) |
| Basal cell cancers, Gorlin syndrome (109400) | *PTCH* | 17 | 33 | 1(1/0/0) |
| Tuberous sclerosis (191100) | *TSC2* | 23 | 132 | 1(1/0/0) |
| Wiskott-Aldrich syndrome (301000) | *WAS* | 2 | 76 | 1(0/1/0) |
| Severe combined immune deficiency (102700, 300400, 312863, 601457, 600802, 602450) | *JAK3* | 17 | 19 | 1(1/0/0) |
|  | *ADA* | 18 | 43 | 1(1/0/0) |
|  | *RAG1* | 47 | 34 | 1(1/0/0) |
| Wilms’ tumor syndrome (194070), WAGR (194072) | *WT1* | 16 | 40 | 1(1/0/0) |
| Hereditary paraganglioma (185470, 115310, 16800) | *SDHD* | 6 | 19 | 1(1/0/0) |
| Endocrine cancer predisposition syndromes (131100) | *MEN1* | 7 | 146 | 1(1/0/0) |
| Total |  | 790 | 2,133 | 141 (125/14/2) |

^a^When the variation is expected to replace one amino acid (AA1) with a different amino acid (AA2)

^b^When the variation is expected to replace one amino acid (AA1) with a termination codon (TeRm)

^c^When the variation is expected not to change the original amino acid (AA1)
